# Supplementary material for: Genomic Instability Signature of Palindromic Non-Coding Somatic Mutations in Bladder Cancer
Source: Cancers (Basel). 2020 Oct 8;12(10):2882. doi: 10.3390/cancers12102882 (PMC7650671; doi:10.3390/cancers12102882)
Supplement: Supplementary file 1 [file cancers-12-02882-s001.zip › cancers-960774-Supplementary materials.pdf]

cancers

Supplementary Materials

Genomic Instability Signature of Palindromic Non-Coding Somatic Mutations in Bladder Cancer.

Sophie Vacher, Voreak Suybeng, Elodie Girard, Julien Masliah Planchon, Grégory Thomson, Constance Le Goux, Simon Garinet, Anne Schnitzler, Walid Chemlali, Virginie Firlej, Diane Damotte, Yves Allory, Maud Kamal, Géraldine Pignot and Ivan Bieche

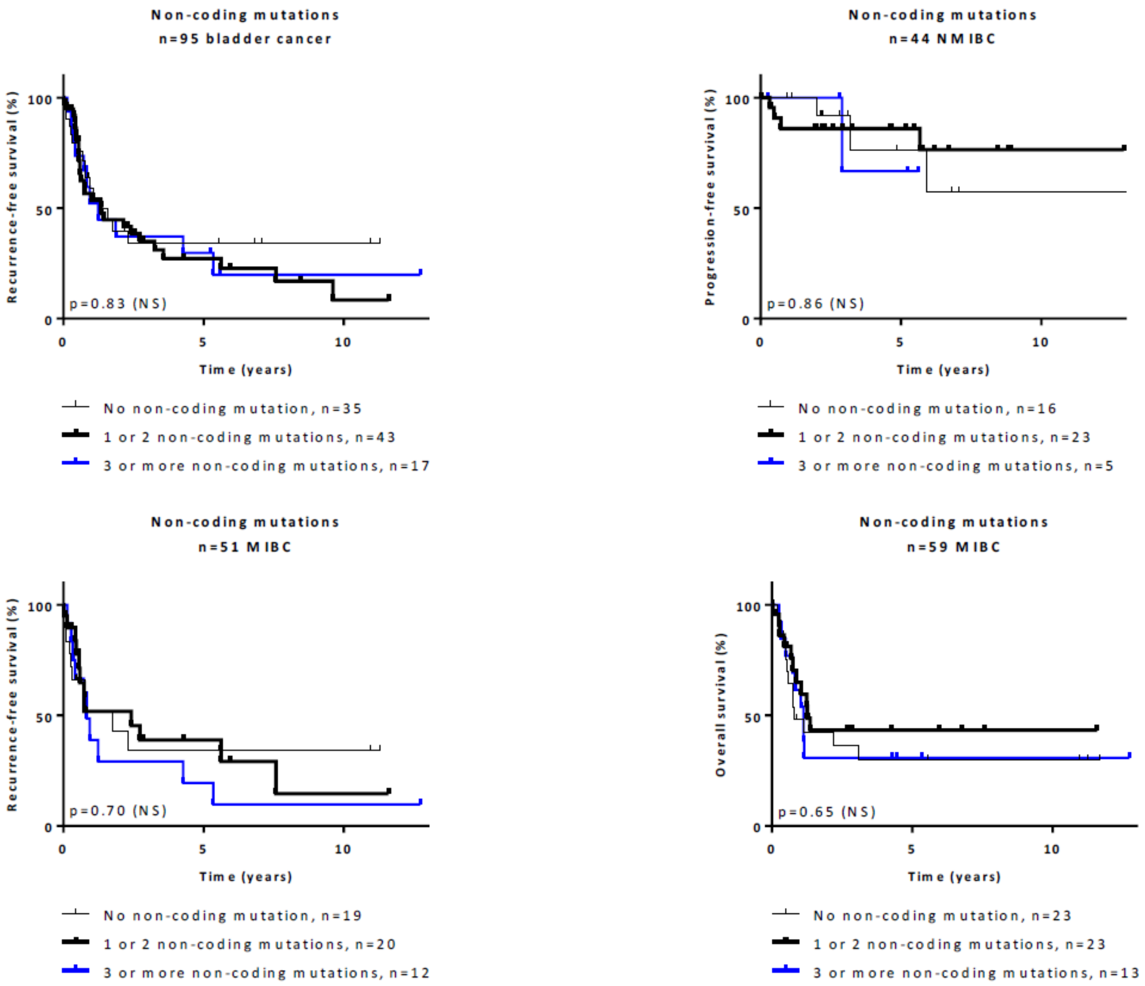

Figure S1. Survival curves of bladder patients according to non-coding mutation status.

Table S1. Primer sequences used for DNA mutation analysis.

Name	Forward Primer (5′–3′)	Reverse Primer (5′–3′)	Product (bp)
	TGAACA core motif identified by Nik-Zainal et al. [13]		
**GPR126**	CCAGTGCATATTTCACATGGACTCT	CAGTAGAGGATGTGTCAATCCTGGA	134
**PLEKHS1**	CTTCCAAGGCTGGGATGATCTA	AAGAAAGTGCCCATAACAGAAATACA	107
**Intron ADM**	GTTGCTTGCTGTGGGAATGATAC	AGCAAGTGCTAAAAAAGGAATCTGT	150
**Chr7:11**	TGCACTACTGTCCACTTCATCAGACT	TGGGCCAGGGTCACTCGTAT	186
**Chr15:96**	GGGCTTGTGTATTTTTTCACTCATC	CTGACTACCCGAAGCAGAAAGAAC	190
**Intron RNF169**	GCCAAATTAAGGAATTTTCCAGAAG	CTGAAGGTTTTTTCCCAATTAACAGA	177
**Chr7:17**	GAGCAAATTTTTGTTGCCACTACAC	TTTGCACTGACCGCAGCACT	127
**Chr10:82**	AGAAAGGCCAGAACTGGGATAGA	AGGAAACTTTTACTCCTCACTCGCT	132

**Chr18:42**	CAAAAGCTGAGATGATGGTCCTG	ATTCTTATCTCACCTGCAAAGCCT	123
**Chr3:82**	GCTTAAAATTGGTGGCAAGT	CCATATGGTTCTAAGATGGCTA	200
	TGAACA core motif identified by* in silico *analysis (present study)		
**GABRG3**	TTTTGTAAAACAACAAGAAATAGTC	GTATAATTTTGTGCAATACGTGA	170
**PCDH18**	CAAGTTTTATTGGAAAGAAGAGTA	TGAGAGATTCCTAATATCTTGTGTA	92
**EYS**	ATTATATTTGGTTTTATGTTTTACAC	TGCTTTAATTTTCTGTAGTCTGA	186
**DAOA**	AGTACTGAAGCTCTACCAAGCTA	GCAATGTTGCTTATTCTCGT	161
**ZNF717**	TGAATTCCTTCTTACTCAATTACT	AGGATCATTTACCTTTCTTACAT	130
**NGLY1**	CTTTTTTAGTATTAGACAATGTGAT	AATTACCAAAAACATACAGAGAT	133
**AP3B1**	GAAACTAAACCATTAGGTATTAGACA	CAGTTATATCCAAAGGCTTGAC	193
**CLVS2**	TTCTGTATTTGGATCTCGTCT	AAATATATGTCTCTCAAAATAACTCT	159
**ZNF621**	TGCCATCTAAGTCCTTCATC	TTTTCAAGTTTGAGAATCACTG	186
**SCHIP1**	GGTTTTCTGTGCCTGGATAC	CTGAATTTCCTTTGAAACACTG	125
**ARAP2**	CACCGTTGAAAAACAGCAG	TCAGGCAATATTTTAACCAGTAG	143
**LOC100422737**	TAACCACATGGTTCAGACATC	CCAATACCGGGTAACTCAG	136
**KIAA1324L**	TATCTGTACTTTCTGTTCACATACT	TTTTGTTATACTTTGTTCATTCA	178
**DKK1**	TGAGGAATATTGTATTCAAATGTG	CTACCTTCCAAATGTTGCTATG	181
**RTN3**	GAGTATATTTTGCAGAAAGACAC	ATAAAGTTAACACAAACTATAAGCTA	165
**OR7E156P**	GCCTATGTGTGAAGATTTTATGA	TGGGACATATGCTTTTAATGAC	113
**LOC644649**	CACCTTTATTGGTCTTATTTAGTACA	CAAATTTTAATGCAATTATTAACTCT	111
**MIR645**	TGATGGAATTGCCTAGTGTGAGTCT	GGACCTCCCCCAACTCTGCT	182
	AGATCA core motif		
**RAD51B**	TGCCAGAAAAACACTTACAC	AGATGAGGATCTCCCACAG	152

Table S2. Sequences of primers used for real-time quantitative RT-PCR.

Gene	Upper Primer (5' to 3')	Lower Primer (5' to 3')	Amplicon Size (bp)
TBP	TGCACAGGAGCCAAGAGTGAA	CACATCACAGCTCCCCACCA	132
APOBEC3A	GACAATGGCACCTCGGTCAAGA	GGGTCCAACTGCAAAGAAGGAAC	137
APOBEC3B	ATTCCTGCACCGCACGCTA	GTCGAAGGACCAAAGGGTCATTA	95
APOBEC3H	CGAGGAAGGCCCTCTTGTGTTA	TTTCTGCATGGCACTTTTTCTTGT	96

Table S3. Mutations in palindromic non-coding sites in the cohort of 103 bladder cancers.

See Excel document.

Table S4. Loci with a TGAACA core motif of palindromic sequences of 9-, 10- or 11-base pair (bp). The 18 loci selected are indicated in blue caracters.

See Excel document.

Table S5. TMB, MSI status, and protein-coding gene alterations in bladder tumors showing high levels of non-coding TGAACA mutations.

Tumor Sample	Number of non-Coding Mutations	TMB	MSI	Number of Non-Synonymous Variants after Filtering	Focal Amplification	Genes with Pathogenic Variants (PV)					
						**Genes**	**Chromosome**	**Transcript**	**NUCLEOTIDIC variant**	**proteic Variant**	**Allelic Ratio**
T331	8	17.7	MSI-H	78	-	CDKN1A	chr6	NM_001291549.1	c.424dup	p.(Glu142GlyfsTer21)	73.68
						TERT	chr5	NA	c.-124C>T	NA	42.52
						KDM6A	chrX	NM_001291415.1	c.1973del	p.(Asn658ThrfsTer19)	40.92
						PTEN	chr10	NM_000314.6	c.492+2T>A	NA	25.62
						RXRA	chr9	NM_002957.4	c.1280C>T	p.(Ser427Phe)	25.34
						ROS1	chr6	NM_002944.2	c.5027G>A	p.(Trp1676Ter)	23.92
						FGFR3	chr4	NM_000142.4	c.1118A>G	p.(Tyr373Cys)	21.85
						PIK3CA	chr3	NM_006218.3	c.1624G>A	p.(Glu542Lys)	21.5
						MLH1	chr3	NM_000249.3	c.958G>T	p.(Glu320Ter)	20.89
						FOXP1	chr3	NM_032682.5	c.1160C>G	p.(Ser387Ter)	20.52
						MLH1	chr3	NM_000249.3	c.2005G>T	p.(Glu669Ter)	19.85
						NSD1	chr5	NM_022455.4	c.1262G>A	p.(Trp421Ter)	16.96
						TP53	chr17	NM_000546.5	c.574C>T	p.(Gln192Ter)	16.8
						PIK3CA	chr3	NM_006218.3	c.277C>T	p.(Arg93Trp)	16.41
						PPM1D	chr17	NM_003620.3	c.859G>T	p.(Glu287Ter)	15.92
						TP53	chr17	NM_000546.5	c.1164_1182del	p.(Glu388AspfsTer28)	13.47
						KMT2A	chr11	NM_005933.3	c.2318del	p.(Pro773ArgfsTer8)	5.78
**T254**	8	12.7	MSS	65	ERBB2	KDM6A	chrX	NM_001291415.1	c.4340dup	p.(Leu1448IlefsTer14)	78.68
						TERT	chr5	NA	c.-124C>T	NA	60.82
						TP53	chr17	NM_000546.5	c.574C>T	p.(Gln192Ter)	43.94
						ERBB3	chr12	NM_001982.3	c.994G>A	p.(Glu332Lys)	37.97
						KMT2D	chr12	NM_003482.3	c.4468G>T	p.(Glu1490Ter)	32.69
						FOXA1	chr14	NM_004496.3	c.412_415dup	p.(Ser139AsnfsTer89)	20.17
**T206**	5	10.4	MSS	93	-	PIK3CA	chr3	NM_006218.3	c.1633G>A	p.(Glu545Lys)	62.62
						ATM	chr11	NM_000051.3	c.7308-1G>C	NA	52.12
						TERT	chr5	NA	c.-146C>T	NA	45.07
						FGFR3	chr4	NM_000142.4	c.1118A>G	p.(Tyr373Cys)	43.56

						ETV6	chr12	NM_001987.4	c.313C>T	p.(Arg105Ter)	40.43
						STAG2	chrX	NM_001042749.1	c.2264del	p.(Lys755ArgfsTer7)	37.95
						PBRM1	chr3	NM_001350075.1	c.237-1G>T	NA	8.74
						ARID2	chr12	NM_152641.3	c.706-1G>T	NA	7.36
						RAD51C	chr17	NM_058216.2	c.1026+1G>T	NA	5.43
**T238**	4	7.5	MSS	52	ERBB2	ERBB2	chr17	NM_004448.2	c.2301C>G	p.(Ile767Met)	79.77
						TP53	chr17	NM_000546.5	c.672+1G>T	NA	38.67
						ASXL2	chr2	NM_018263.4	c.760G>T	p.(Glu254Ter)	25,0
						ARID1A	chr1	NM_006015.5	c.5393C>G	p.(Ser1798Ter)	23.53
						ERBB2IP	chr5	NM_018695.3	c.131_135del	p.(Phe44Ter)	18.62
						NIPBL	chr5	NM_015384.4	c.2509C>T	p.(Gln837Ter)	15.79
**T272**	0	5.6	MSS	38	ERBB2	RB1	chr13	NM_000321.2	c.1694C>A	p.(Ser565Ter)	34.5
						TERT	chr5	NA	c.-124C>T	NA	31.82
						TP53	chr17	NM_000546.5	c.536A>G	p.(His179Arg)	27.86

MSI: Microsatellite Instability; MSS: Microsatellite Stable; MSI-H: Microsatellite Instability High; NA: non applicable

Table S6. Associations between TGAACA core motif mutational signature and mRNA expression levels of immune-related genes.

Gene	Gene ID	Aliases	Group 1 No non-coding mutations (*n* = 39)			Group 2 One or two non-coding mutations (*n* = 46)			Group 3 Three or more non-coding mutations (*n* = 18)			*p*-Value *
			Median	Min	Max	Median	Min	Max	Median	Min	Max	
**Immune cell population genes**												
CD2	914		**1.00**	0.01	7.47	**0.56**	0.06	6.81	**0.67**	0.16	2.33	0.39 (NS)
CD3E	916		**1.00**	0.08	9.68	**0.65**	0.06	7.19	**0.87**	0.15	2.78	0.23 (NS)
CD4	920		**1.00**	0.17	5.36	**0.73**	0.11	4.11	**0.99**	0.13	1.97	0.11 (NS)
CD8A	925		**1.00**	0.05	29.1	**0.65**	0.07	13.5	**1.22**	0.15	8.95	0.68 (NS)
CTLA4	1493		**1.00**	0.00	8.63	**0.58**	0.00	5.65	**1.27**	0.08	3.10	0.32 (NS)
FOXP3	50943		**1.00**	0.12	6.67	**0.78**	0.08	5.64	**0.94**	0.07	3.43	0.22 (NS)
XCR1	2829		**1.00**	0.09	10.8	**0.73**	0.05	14.2	**0.97**	0.00	15.3	0.15 (NS)
MERTK	10461		**1.00**	0.24	3.55	**1.07**	0.27	2.41	**0.98**	0.14	3.11	0.90 (NS)
PTPRC	5788	*CD45*	**1.00**	0.02	7.18	**0.58**	0.02	6.14	**0.97**	0.13	2.47	0.19 (NS)
MS4A1	931	*CD20*	**1.00**	0.00	28.6	**0.43**	0.00	39.9	**1.45**	0.00	25.2	0.79 (NS)
NCAM1	4684	*CD56*	**1.00**	0.00	108.4	**1.11**	0.00	47.3	**1.41**	0.22	64.4	0.84 (NS)
PDGFRB	5159		**1.00**	0.07	7.29	**0.50**	0.08	6.42	**0.87**	0.07	3.23	0.17 (NS)
PECAM1	5175		**1.00**	0.14	6.14	**0.66**	0.10	2.83	**0.83**	0.19	2.74	0.14 (NS)
**T cell activation genes**												
PRF1	5551		**1.00**	0.09	16.8	**0.47**	0.05	10.4	**0.83**	0.10	3.08	0.34 (NS)
GZMA	3001		**1.00**	0.03	31.7	**0.43**	0.01	9.42	**1.03**	0.12	4.65	0.11 (NS)
GZMB	3002		**1.00**	0.01	40.8	**0.41**	0.02	20.1	**1.51**	0.02	11.1	0.38 (NS)
**Checkpoint T cell genes**												
CD28	940		**1.00**	0.01	5.45	**0.64**	0.02	4.89	**0.87**	0.08	1.61	0.062 (NS)
ENTPD1	953	*CD39*	**1.00**	0.10	4.13	**0.66**	0.10	3.55	**0.75**	0.12	2.32	0.28 (NS)
NT5E	4907	*CD73*	**1.00**	0.05	8.6	**0.55**	0.06	4.58	**0.39**	0.16	6.06	0.061 (NS)
CD96	10225		**1.00**	0.07	4.88	**0.82**	0.06	3.21	**0.78**	0.03	5.67	0.76 (NS)
TIGIT	201633		**1.00**	0.07	12.1	**0.74**	0.06	8.35	**1.64**	0.13	3.62	0.40 (NS)
CD226	10666		**1.00**	0.02	5.13	**0.46**	0.04	3.18	**0.70**	0.10	2.32	0.081 (NS)
TNFRSF14	8764		**1.00**	0.35	3.06	**0.94**	0.24	1.87	**0.81**	0.07	2.31	0.23 (NS)
TNFRSF18	8784	*GITR*	**1.00**	0.09	8.99	**1.01**	0.03	11.33	**0.86**	0.03	2.79	0.61 (NS)
TNFRSF4	7293	*OX40, CD134*	**1.00**	0.09	8.03	**0.66**	0.08	4.24	**1.22**	0.17	3.00	0.37 (NS)
CD27	939	*TNFRSF7*	**1.00**	0.00	17.4	**0.68**	0.00	10.7	**1.14**	0.10	5.02	0.44 (NS)
TNFRSF9	3604	*CD137, 4-1BB*	**1.00**	0.00	13.7	**0.81**	0.00	16.75	**1.37**	0.12	5.25	0.59 (NS)
HAVCR2	84868	*TIM3*	**1.00**	0.08	14.2	**0.67**	0.05	7.03	**1.04**	0.11	6.17	0.31 (NS)
ICOS	29851		**1.00**	0.00	8.44	**0.50**	0.02	5.36	**0.80**	0.10	1.74	0.15 (NS)
LAG3	3902		**1.00**	0.04	64.8	**0.95**	0.04	19.39	**1.17**	0.08	14.4	0.94 (NS)
PDCD1	5133		**1.00**	0.00	9.75	**0.66**	0.00	10.64	**1.25**	0.13	4.04	0.66 (NS)
**Checkpoint tumor cell genes**												
IDO1	3620		**1.00**	0.05	109.3	**0.89**	0.03	121.8	**1.52**	0.08	78.9	0.93 (NS)
CD80	941	*B7-1*	**1.00**	0.00	25.2	**0.73**	0.03	5.55	**1.45**	0.09	9.01	0.56 (NS)
CD86	942	*B7-2*	**1.00**	0.07	9.99	**0.80**	0.07	6.02	**1.36**	0.27	5.76	0.55 (NS)
CD276	80381	*B7-H3*	**1.00**	0.44	3.14	**1.00**	0.32	3.20	**1.30**	0.49	5.01	0.82 (NS)
LGALS9	3965	*LGALS9*	**1.00**	0.07	7.68	**1.10**	0.16	5.66	**1.34**	0.10	2.29	0.48 (NS)
CD274	29126	*PDL1*	**1.00**	0.05	24.1	**0.89**	0.06	18.1	**1.49**	0.09	7.78	0.84 (NS)
PDCD1LG2	80380		**1.00**	0.03	17.2	**0.58**	0.04	17.7	**1.15**	0.10	4.17	0.30 (NS)
ICOSLG	23308		**1.00**	0.20	3.31	**0.80**	0.18	3.55	**1.29**	0.39	4.81	0.22 (NS)
PVR	5817	*CD155*	**1.00**	0.26	3.00	**0.93**	0.00	3.40	**0.99**	0.23	3.01	0.78 (NS)
PVRIG	79037	*CD112R*	**1.00**	0.21	6.71	**0.56**	0.21	5.05	**0.96**	0.25	4.06	0.15 (NS)
TNFSF4	7292	*OX40L*	**1.00**	0.00	44.0	**1.42**	0.09	27.9	**4.66**	0.00	39.2	0.75 (NS)
**Inferferon inducible genes**												
CXCL10	3627		**1.00**	0.02	269.9	**1.20**	0.02	85.7	**2.60**	0.04	44.6	0.69 (NS)
RSAD2	91543		**1.00**	0.24	42.0	**3.21**	0.11	22.5	**3.48**	0.31	87.7	**0.044**
IFI44L	10964		**1.00**	0.04	31.2	**1.91**	0.04	21.3	**1.67**	0.07	33.6	0.15 (NS)
IFI6	2537		**1.00**	0.11	16.6	**1.96**	0.05	16.5	**1.90**	0.19	47.0	**0.045**
IFIT1	3434		**1.00**	0.11	18.1	**1.16**	0.08	15.8	**1.03**	0.09	39.1	0.26 (NS)
IRF8	3394		**1.00**	0.06	7.88	**0.82**	0.02	6.47	**1.23**	0.15	3.04	0.37 (NS)
MX1	4599		**1.00**	0.12	15.5	**1.52**	0.18	17.4	**1.14**	0.11	30.1	0.21 (NS)
OAS1	4938		**1.00**	0.13	4.16	**1.27**	0.36	9.48	**1.47**	0.02	5.24	**0.035**

IFI27	3429		**1.00**	0.10	34.1	**3.64**	0.12	50.1	**1.03**	0.24	26.6	**0.015**
G1P2	9636	*ISG15*	**1.00**	0.12	11.9	**1.53**	0.09	7.78	**1.75**	0.18	27.3	**0.025**
**Major histocompatibility complex genes**												
HLA-A	3105		**1.00**	0.14	7.81	**0.98**	0.03	5.85	**0.91**	0.25	6.66	0.32 (NS)
HLA-B	3106		**1.00**	0.12	36.1	**1.07**	0.12	13.4	**0.73**	0.12	13.9	0.52 (NS)
HLA-C	3107		**1.00**	0.00	5.25	**0.38**	0.00	4.85	**0.12**	0.00	8.23	0.48 (NS)
HLA-DRA	3122		**1.00**	0.02	7.09	**0.50**	0.02	4.98	**0.82**	0.10	3.07	0.14 (NS)
HLA-DRB1	3123	*HLA-DRB*	**1.00**	0.08	6.16	**0.65**	0.02	5.36	**0.88**	0.11	3.71	0.37 (NS)

* Mann-Whitney test (no *vs.*1 or more non-coding mutations).

© 2020 by the authors. Licensee MDPI, Basel, Switzerland. This article is an open access article distributed under the terms and conditions of the Creative Commons Attribution (CC BY) license (http://creativecommons.org/licenses/by/4.0/).
